# Ethical concerns with the use of intelligent assistive technology: findings from a qualitative study with professional stakeholders

**DOI:** 10.1186/s12910-019-0437-z

**Published:** 2019-12-19

**Authors:** Tenzin Wangmo, Mirjam Lipps, Reto W. Kressig, Marcello Ienca

**Affiliations:** 1 Institute for Biomedical Ethics, University of Basel, Basel, Switzerland; 2 Faculty of Medicine, University of Basel, Basel, Switzerland; 3 Chair of Geriatrics, University of Basel; Chief Medical Officer, University Department of Geriatric Medicine Felix Platter, Basel, Switzerland; 4 Health Ethics & Policy Lab, Department of Health Sciences and Technology, ETH Zurich, HOA H 17, Hottingerstrasse 10, 8092 Zurich, Switzerland

**Keywords:** Dementia, Assistive technology, Artificial intelligence, Robotics, Ethics, Care, Autonomy, Justice

## Abstract

**Background:**

Advances in artificial intelligence (AI), robotics and wearable computing are creating novel technological opportunities for mitigating the global burden of population ageing and improving the quality of care for older adults with dementia and/or age-related disability. Intelligent assistive technology (IAT) is the umbrella term defining this ever-evolving spectrum of intelligent applications for the older and disabled population. However, the implementation of IATs has been observed to be sub-optimal due to a number of barriers in the translation of novel applications from the designing labs to the bedside. Furthermore, since these technologies are designed to be used by vulnerable individuals with age- and multi-morbidity-related frailty and cognitive disability, they are perceived to raise important ethical challenges, especially when they involve machine intelligence, collect sensitive data or operate in close proximity to the human body. Thus, the goal of this paper is to explore and assess the ethical issues that professional stakeholders perceive in the development and use of IATs in elderly and dementia care.

**Methods:**

We conducted a multi-site study involving semi-structured qualitative interviews with researchers and health professionals. We analyzed the interview data using a descriptive thematic analysis to inductively explore relevant ethical challenges.

**Results:**

Our findings indicate that professional stakeholders find issues of patient autonomy and informed consent, quality of data management, distributive justice and human contact as ethical priorities. Divergences emerged in relation to how these ethical issues are interpreted, how conflicts between different ethical principles are resolved and what solutions should be implemented to overcome current challenges.

**Conclusions:**

Our findings indicate a general agreement among professional stakeholders on the ethical promises and challenges raised by the use of IATs among older and disabled users. Yet, notable divergences persist regarding how these ethical challenges can be overcome and what strategies should be implemented for the safe and effective implementation of IATs. These findings provide technology developers with useful information about unmet ethical needs. Study results may guide policy makers with firsthand information from relevant stakeholders about possible solutions for ethically-aligned technology governance.

## Background

There were approximately 50 million persons living with dementia in 2017 and this prevalence is estimated to reach 75 million by 2030 [1], despite decrease in incidence of dementia [2]. The global cost of dementia was above $1 trillion in 2018 [1, 3], double the cost estimated in 2009 [4]. As no effective treatment is currently available for most neurodegenerative causes of dementia such as Alzheimer’s disease, dementia represents a primary public health concern [5] whose impact affects the physical and emotional wellbeing of affected patients and their family members, as well as the financial sustainability of the health care system [2, 6].

The developments taking place in the field of intelligent assistive technologies (IATs) are expected to mitigate the caregiving burden and high costs associated with caring for persons living with dementia [7–9]. The IAT umbrella term is used to refer to a variety of technologies leveraging on computing capabilities, robotics and machine intelligence for assistive purposes [10]. Recent studies have mapped and assessed the growing spectrum of available IATs to aid persons with dementia and/or their caregivers. A first review from 2009 [11] identified 58 IATs comprising primarily cognitive aids to support memory, aphasia and agnosia; physiological sensors to detect vitals and falls; environmental sensors to detect movement; and advanced security systems. A more recent systematic review updated the list of available IATs and identified 539 IATs with possible applicability for older patients with dementia [10]. These IATs include devices for assistance in the completion of activities of daily living (ADL), systems for cognitive and emotional assistance, health and behavioral monitoring, social interaction and engagement, remote communication, emergency alarm and mobility aids. Data breakdown by study design indicates that only 40% of current IATs are designed through user-centered approaches. That is, they involved end-users in the development phase to achieve clinical validation, ex-ante technology assessment and iterative calibration based on end-users’ needs.

IATs for people with dementia also raise substantive ethical challenges [12–16]. The reason for that stems from the fact that these technologies are designed for vulnerable older individuals with physical frailty and cognitive disability, who often lack the capacity to consent to their use. Furthermore, IATs typically collect large volumes of differently structured user data that can be processed at high velocity, rendering the IAT ecosystem a “big data” ecosystem [17, 18]. This large-scale information processing might involve sensitive data including personally identifiable data, such as a person’s medical information or behavioural video-recordings, or might enable retrospective information retrieval from de-identified datasets. Finally, several IATs operate in close proximity to the patient’s body (e.g. wearables) and/or involve varying degrees of artificial intelligence, hence raising ethical challenges in terms of algorithmic transparency [19], agency and responsibility [20]. A systematic review has observed that 67% of current IATs for dementia are designed in absence of explicit ethical assessment [21]. This raises concerns about the ethical viability of using these technologies among vulnerable individuals. Among the portion of IATs that did include ethical assessment, primary attention was devoted to respecting the autonomy of patients, preventing harm (non-maleficence) and promoting overall good (beneficence) [21]. Ethical concerns such as ensuring fair technology access (distributive justice) and preserving by design the privacy of end-users and their data appear underrepresented. The focus on autonomy is unsurprising considering that the need for IATs is often predicated upon the value of empowering older adults with dementia by increasing their independence and prolonging their independent living [12, 15]. Two previous reviews also highlighted the importance of issues of informed consent, autonomy, privacy, data security and affordability as key ethical concerns when using IATs [22, 23] among people with dementia. Other ethical concerns discussed in the literature include stigma, social isolation, lacking user-engagement in the design of the technology as well as the ethical dilemma about whether IATs would and should replace human care [24].

Investigating the views and needs of both patients and relevant stakeholders at the cross-section of technology development and healthcare is considered a useful way to prospectively assess the practical, technical, clinical and ethical challenges associated with IATs [25]. However, only a few empirical studies have captured the opinions of involved stakeholders concerning their experiences and attitudes towards IATs. For example, Kramer (2014) investigated the views of caregivers of people with dementia in Germany and revealed the presence of an “information gap” between developers and end-users, which is likely to affect adequate adoption among end-users [26]. Using a similar approach, Ienca and colleagues [25] interviewed healthcare professionals in three European countries, highlighting the perceived significance of ensuring user-centeredness when designing IATs. Another study from the UK explored the views and experiences of people with dementia, their carers and General Practitioners about use of IATs in dementia care [27]. These studies observed that stakeholders are aware of available IATs and their applicability but underlined existing barriers that prevent their widespread uptake among end-users. The barriers include the relatively high costs of IATs and the lack of knowledge about when these technologies should be used to maximize health outcomes [28]. Also noted are issues of access to IATs, affordability and ethical use [25, 28]. Finally, one study assessed IATs for behavioral monitoring [29] and concluded that carers generally find the use of IATs for the home surveillance of people with dementia ethically acceptable, despite acknowledging the existence of important privacy issues.

In light of the limited corpus of empirical studies delineating the ethical issues associated with the use of IATs in dementia care, it is important to understand the views of different stakeholders involved in the development and management of technology-assisted elderly and dementia care and proactively consider the ethically relevant issues emerging in this domain. Thus, we addressed this research gap by conducting a study involving health professionals and researchers (hereafter, professional stakeholders) working in dementia and psychogeriatric care with the aim of identifying critical ethical concerns perceived in relation to the use of IATs for the care of older people with dementia.

## Methods

### Participant recruitment

Health care personnel (medical doctors, nurses and nursing home managers) and researchers in the fields of geriatrics, psychiatry, neurology, neuropsychology, gerontology and nursing participated in this study. We recruited participants from Switzerland, Germany and Italy using purposive sampling method. We searched for prospective participants using institutional homepages and ensured that we have a good balance of public and private health institutions and varying experience (see [25]). One author *(MI)* with training in qualitative research methodology sent emails to prospective participants explaining them the purpose of the study, its methodology, risks and benefits, and the informed consent form. 24 potential participants were contacted for participation, 21 of which agreed to participate in an interview. One participant subsequently withdrew from the study after initial enrollment due to health issues. Upon positive response, an interview date and time was scheduled with 20 participants. Prior to data collection, participants signed a written informed consent form. Table 1 presents a detailed overview of the study population.

**Table 1:** Demographic information of the study participants (*N* = 20)

Country	n (%)
Switzerland	10 (50)
Italy	6 (30)
Germany	4 (20)
Professional experience	
Gerontology	1 (5)
Geriatrics	3 (15)
General practitioners	1 (5)
Neurology	3 (15)
Neuropsychology	4 (20)
Nursing	1 (5)
Nursing home management	1 (5)
Psychiatry	6 (30)
Gender	
Male	11 (55)
Female	9 (45)

### Data collection

Before the interview, we created a list of topics to guide the discussions. These included among others (A) participants’ expectations, needs and perceptions surrounding clinical application of IATs; (B) their experiences with the use of IATs and interactions with IAT designers, developers and other stakeholders; (C) their perspectives on governance and management of IATs; and (D) participants’ expectations about the future of dementia care in a digital world. We adapted the interview guide as the interviews progressed and included probing questions wherever necessary. The interview guide was pilot-tested internally prior to data collection (see Additional files 2 and 3).

One author *(MI*) carried out the interviews in German, English or Italian based on the preference of the study participants. Of the total 20 interviews, 15 were completed face-to-face at a place agreed upon with the participant and the remaining five were conducted using video call. During these interviews, no other person except the interviewer and the interviewee were present. The interviews were between 21 to 55 min long (average 33 min). All interviews were audio recorded and transcribed verbatim in the language of the interview with the assistance of the f4transkript software v2. All participants were given the opportunity to check the transcripts. Only two participants wished to do so. In both cases, the transcript was approved without modification. Audio files were iteratively assessed to determine whether theoretical saturation was reached by the time of completion of the data collection. This assessment confirmed that data saturation was reached.

### Thematic analysis

Data analysis for this paper occurred in two phases. In the first phase, two authors read and thematically coded all transcribed interviews using the MAXQDA Standard software for qualitative data analysis (version for Windows). In this phase, we coded macro-thematic families, not restricted to the sole ethical dimension but also addressing social, financial, and technical considerations. Another paper [25] highlights results from this coding. In the second phase, ML extracted the ethics-related coded sections from this first-level coding. To ensure that all data related to ethical concerns were taken into consideration, TW read entire transcripts to gather and contextualize participants’ opinions on ethical concerns related to IATs. She thus added sections that were not included in the ethics-related codes. The relevant data for this paper (48 pages of single spaced data) were then coded again inductively using descriptive thematic analysis [30]. That is, we recoded these codes into more specific ethics-related themes and sub-themes.. In terms of coding validity, the two coding phases are methodologically congruent and consistent, but differ in focus and degree of granularity.

Our analysis resulted in the development of four issues within this rubric of ethics: (1) inability of persons with dementia to decide for themselves; (2) data management; (3) affordability of IATs which raise the issue of who will ultimately have access to these IATs; and (4) the imperative to ensure human contact when caring for persons with dementia (Table 2). The first two themes incorporated a few sub-themes.

**Table 2:** Themes and sub-themes related to ethical concerns

1. Mental (in)capacity	2. Data management	3. Affordability – Distributive justice	4. Human Contact is crucial
a. Informed consent	a. Data access		
b. Advanced directives	b. Data sharing		
c. Deception			

To exemplify our findings, we have used direct quotes from our participants. These quotes were translated to English by ML and another assistant, both fluent in German and English, and MI translated Italian quotes to English. These translated texts were checked for meaning by the first and the last author. Where necessary, tacit or implicit information was made explicit by the authors and presented in square brackets to make the sense clearer to the reader.

In light of the overall purpose of the project and in accordance with the Human Research Act, this study received waiver of ethical approval from the competent cantonal ethics commission in Switzerland.

## Results

### Mental (in)capacity

#### Informed consent

In light of the progressive cognitive deterioration caused by dementia, interviewees often noted that older adults with dementia might have a reduced or even entirely compromised ability to decide for themselves about the use of IATs. In such cases, it was argued that the surrogates should take their decisions based on good knowledge of what the IAT does and what the patient would have wished. In case the patient had given prior indications about future choices, this will should be prioritized.

*If there is someone who has the role of a proxy and has the power of attorney, he/she can decide [for the use of IATs] even if the [patient] is already severely [mentally] affected [by aging or dementia] and can’t decide for him/herself. (…) you should not deal so frivolously with the matter: it’s important to be aware of the fact that by monitoring a person you are observing that person. For that [observation], the person must have given their consent, only then it is okay. (P 12).*

*In doubt, it is always the relative [who is] the legal guardian, who has to make the decision. Purely out of legal perspective, his/her decision should correspond with the will of the patient since they would have talked about it. (P 14).*

#### Advance directives

To overcome the difficulties associated with obtaining consent from older adults with dementia (especially those with moderate to advanced dementia), interviewees often addressed the use of advance directives as a way to understand the expressed wishes of the older person. Several interviewees critically discussed whether asking older persons to deliberate on their wishes and future choices at a time when they are still able to do so, could help respect their autonomy throughout the disease trajectory. Most interviewees favored the use of such an advance directive. They suggested that knowing older adults’ decisions about IAT use before the progression of their cognitive impairment would be a clinically effective and ethically sound way to empower them and respect their (future) autonomy.

*An alternative is that, today as a healthy person I can say that ‘either I get lost in the forest and will not be found or I am happy if somebody finds me’. When I am in that circumstance [*i.e. *have dementia] that I can’t make sound judgement – I could have stated earlier in my living will or power of attorney. (P 1).*

*We do a lot of different types of studies where [we use] some ahead of time approach you mentioned like in the future, that they are still, that they can still, their previous willingness, they can participate in future studies even though they might not have the cognitive capacities to provide informed consent. The studies that we did, that we published with the prototype locating system that was people who were cognitively fit to give their written informed consent. (P 17).*

Equally relevant to the theme of decision-making was the proposal of using behavioral observation to respect the current ‘autonomy’ of older persons with moderate-to-advanced dementia. In fact, participants noted that while advance directives are a useful decisional instrument, people with dementia may explicitly revoke their initial consent at a later stage or implicitly show signs of distress. This led interviewees to propose a Best Interest standard (BIS) to revoke past consent. This solution included continuously monitoring possible signs of agitation or discomfort in older adults with moderate-to-advanced dementia who are using IATs.

*Unfortunately, people with dementia can neither decide nor make sound judgments – they can only decide for the present moment: “I don’t like this robot.” Yet, what if the robot doesn’t notice it and perhaps reacts wrongly? On the either hand, if the patient with dementia dislikes the robot, it is also a decision that must be respected. (P 6).*

#### Deception

Within the framework of autonomy, participants also discussed whether IATs might, under certain circumstances, qualify as a form of deception. For example, interviewees expressed the fear that human-shaped or pet-shaped socially assistive robots might implicitly deceive older adults with dementia and be erroneously perceived as real humans or pets (and hence may not depict negative behaviors when using IATs). It was observed, that even when zoomorphic robots are not explicitly presented to the patients as real pets, their pet-like shape could induce patients to perceive and treat them as biological animals, hence be inherently deceptive. A few participants saw this alleged risk of deception in opposition to professional ethics and even associated with the notion of “dignity”.

*I can imagine that in the daily interactions pet-shaped or baby-shaped robots will be used by older persons with dementia and perceived as real. I have seen this myself. The question then is, is it deception if the pet-shaped or baby-shaped robot is perceived as real... a real being instead of a robot? (P 6).*

*Maybe it is simply a subjective opinion …. I worry about deceiving people. I am an unbelievably honest doctor. By using robots [I feel] I somehow deceive humans. (P 8).*

*I do not know if you could test and find a difference [whether using pet-robots is perceived as a deception by the patient or not]. Yet, for me … yet, for me it is all about dignity. OK, if a person with dementia has a stuffed-toy … then why not? Many older persons have animals at home, who they love, no? But with the robben [Paro robot], we present it [to the patient] as something else, no? (P 8).*

In response to the same problem, however, an interviewee implied that the moral importance of preventing deception should be subordinated to the moral obligation to improving the patients’ wellbeing. Accordingly, it was argued that if a pet-shaped socially assistive robot can effectively contribute to ensure the wellbeing of the patient (e.g. mitigating their agitation), then the moral obligation to ensure the wellbeing of the patient should have priority over the possible risk of deception.

*There are also cats … wonderful… I have seen them as well … and I have, they [the pet-robots] come close and you automatically feel the impulse to stroke them. It has a beautiful fur and then … then they purr or make other sounds … Therefore, I don’t see any ethical problem … I also don’t think you deceive humans … if I observe a small child playing with a small car on a lawn, you could also say*: *“Yes, you are betraying this child”- It doesn’t matter… The main thing is that the human feels comfortable … of course you shouldn’t use these robots as a replacement of humans … that’s clear for sure, but that’s also absolutely not the idea. (P 1).*

### Data management

Most participants discussed the issue of data management in order to ensure data protection when using IATs such as activity-tracking wearables and ambient sensors, and transmission of such information to third-party services. Thus, two issues were significant within this thematic family: access to data and data sharing that enveloped privacy and autonomy rights of persons with dementia.

#### Data access

Specifically, to ensure greater privacy, some interviewees suggested that data collection should be restricted to what is necessary for clinical purpose and called for clearer conditions for data access and storage in order to give patients greater control over their personal data. In determining such conditions for data access, participants suggested that the patients themselves should be entitled to some degree of data ownership rights. However, a critical discussion of what data ownership might mean in the context of IATs for dementia remained largely unelaborated.

*In the case of around-the-clock tracking, there is especially the need to take into account the autonomy of the patients with dementia. The patient must have the possibility to shut off the device temporarily if he does not want to be observed. That is important. Otherwise, it is a bit like “big brother is watching you” and I believe that is an ethical challenge. (P 7).*

*In general, it is that the patients themselves do not foresee the full range of consequences and their [technologies’] impact. So, it’s mainly the caregivers who should make these decisions. If you take a GPS system, for example, the option for many of these systems is that the device sends an alert when the person oversteps a certain range ... then the alert signal goes on. And this is also between a patient and a caregiver, a quite borderline privacy question …. there are also a lot of patients living in nursing homes and if devices like these would be used in a more professional setting or at hospitals, I would be even more skeptical about how these technologies could be used. And if it’s always in the best interest of the patient. (P 13).*

*You just mentioned data handling. I believe this is very important and very interesting because data handling… the use of data, data processing… basically who owns the data, where will the data be saved etc*. *That is a debate which should be addressed urgently … Realistically speaking, we as people who do not work in a data center, we cannot be in every data center every time. We do not know where our data is and what happens with it. That is simply a fact. We must believe in what we are told – and of course from the ethical standpoint, I mean an ethical critical point is that when collected data is being used against the patient, for example by health insurance … (P 14).*

#### Data sharing

A few participants mentioned that data transmission should occur within a closed system in order to protect individual privacy. Also mentioned was that data subjects would agree to share relevant information with third parties such as healthcare professionals and irrespective with whom data is shared. Nonetheless, obtaining consent from the data subjects was advocated as a necessary requirement and non-negotiable principle.

*«Wearables» or implants or whatever, it doesn’t matter… I give my data and of course, I agree that if a health indicator exceeds a critical value, my general physician or whoever else should be informed and then he can call me… Sure, that is fine. But if this information is then used by pharma companies for example, or by others… It just needs to be clear who uses this data. It needs to be clear, that is a solvable problem. It does not matter to me if a pharma company uses it. I would be ok with it but someone else might not be. Then one needs to be able to decide. (P 1).*

### Affordability – Distributive justice

The interviewer posed a question related to costs of IATs and asked whether costs could result in social disparity. Many participants agreed that IATs are usually expensive as they involve costly hardware and proprietary software, which also means that IATs are not available equally to all those who need it. However, a few interviewees also stated that such social inequality is not a new problem but inherent to new technology. Electricity, personal computing and mobile telephone were provided as examples of technologies, which were originally marketed at high prices but later became increasingly affordable and widely used across all socio-economic strata. This observation raised the consideration that while IATs are likely to create socio-economic disparities between upper and middle-to-lower class seniors, such disparities are likely to equal out with time.

*Well, one of the things would be the eventual price range. Because if it’s a device that turns out to be very useful but a lot of patients wouldn’t be able to afford it, I don’t think that would be making much sense looking into that further. And the other thing is... mmm... (P 13).*

*So I believe, based on my experience with a research project that I worked on recently, that technology development always implies that at a certain point a social… I leave out the «social»… an inequality between different groups occurs. And there is always, there will always be situations in technology development in which a group of devices… a category of devices is on the market and the people who choose it have an advantage compared to those who don’t choose it. Therefore, there is* per se *an inequality between these two groups. Like you just explained. So first my answer: Yes. The question here is how these systems become established and how they might sooner or later become available as everyday technology for everyone. Examples [of this transition] are electricity, telephones… (P 14).*

While interviewees predicted a trajectory of progressive cost-reduction in analogy with other technologies, some also suggested that welfare policies could accelerate the uptake of IATs across all socioeconomic strata and ensure a fair access to this technology. In particular, one interviewee argued that an inclusive social insurance policy in which IATs are partly or entirely reimbursed under basic health insurance plans, may effectively facilitate their widespread use, overcome translational barriers, and ensure a fairer access.

*And also in Germany I hear this from my colleagues that have more experience than me. They say that there would - barrier is that there is - help insurance companies, that would be a huge advantage, they would take over the costs of the locating systems, assistive technologies. That they would able to promote them and take over some of the costs. (P 17).*

Besides insurance and reimbursement, one interviewee hypothesized that the cost of IATs could be reduced by promoting open development at both hardware and software level. Open-source hardware and software development could possibly curb the costs of IATs. However, ethical obligations to open development could be in opposition to the previously described ethical obligation to ensure data protection.

*Open access makes it possible that the [latest] products cost much less than the first products on the market. For example, the greater access through “open access” can be made with program codes … However, when speaking of “open access”, we are also raising a problem of liability. A company that provides open access platforms or something like that, then it also faces a critical problem, the problem of many hands, which could cause, for example, problems in terms of data security. Here again, we are talking about dementia: from my point of view this [open access] can get quite difficult to be accepted by insurance. Who takes the liability for it in the end? (P 14).*

### Human contact is crucial

The final recurrent ethically relevant theme was the widespread belief that IATs, at least in their current level of technological sophistication and market maturity, should complement but not replace human-delivered care. This belief appeared to be primarily motivated on ethical grounds, particularly on the idea that human contact and empathy are essential features of clinically effective and morally acceptable care.

*For us I am not sure whether this works and if it is good. I do not see that technology would help a lot, because it [our work] is about personal contact, about empathy and human company and so on. It is about deeply emotional things and there I do not see how technology could replace it. (P 9).*

*Definitely integration but not replacement of human care. I mean nowadays in hospitals, in nursing homes, we already know that many interactions are too brief and do not provide patients with what they really need at the human level... The belief is that, in these settings, if you watch the patients and give them food 3 times a day, then you are taking care of someone. But that’s just the beginning. When you interact with someone. When you talk, when you touch someone. So the more we replace these things, this kind of information flow, this interaction... if we really replace that with technology, patients will be deprived even more of what they need most. (P 13).*

*For example, to lift heavy patients such instruments are very very helpful, for sure, but they can never replace this humanness in care. I find this development [technological use in care provision] a bit ambivalent. There is this great ability to facilitate everyday life but if it ends up fully replacing human care, then there is a loss of human closeness, a loss of empathy and a loss of emotional exchange. (P 4).*

Other interviewees, in contrast, grounded their sceptical belief in financial considerations related to the current costs of IATs and the previously described moral problem of deception.

*P 8: Being human is not replaced by technology. For example, I extremely dislike this seal [referring to Paro]. I have tested it. I find it extremely funny but for me, one should not deceive people. If one does any dog therapy, that costs. This seal is very expensive, it costs…*

*Interviewer: 5000 dollars.*

*P 8: Yes, exactly. For this [amount of money] I can afford many therapy units with a real dog, right?*

Several interviewees highlighted that while IATs cannot and should not replace care, their adequate introduction could actually promote and enhance – rather than threaten - human-delivered care. This prediction was grounded in the belief that successful IATs could free health professionals, especially nurses, from administrative and physical tasks, hence allow them to invest more time in the social and emotional support of patients.

*We have therapy groups. We have two residents, they need the dolls the whole day, and it is their baby. It works well and if it is good for the resident, why not? But it doesn’t replace human care. It is an added value but not a replacement. So it would be important for me, well generally I think it needs to be ensured that the nursing staff has enough time to spend with the person, with the dementia patient, and care for him. One should be able to relieve caregivers from all the administrative stuff. That has nothing to do with technology. (P 3).*

Finally, a moral argument was advanced stating that while technology-mediated replacement of human-delivered care is a suboptimal choice, it is still preferable to no care at all.

*Yes, I think that [technologies] is the last option. I think after everything, if the alternative is that the people have no care at all then it is of course a good replacement to take care of different personal needs … (P 4).*

## Discussion

The interviewees’ focus on patient autonomy and the problem of obtaining adequate consent from people with dementia largely reflects the concerns of IAT researchers [21]. Furthermore, it highlights the practical and normative challenges emerged in the scientific and bioethical literature [15]. The challenge of obtaining consent from older adults with dementia and thereby respecting their autonomy has been widely discussed in relation to pharmacological interventions [31, 32]. Our findings illustrate that many of these challenges also apply to IATs. These include the use of proxy consent and advance directives to obtain consent from patients with diminished or lost mental capacity as well as behavioral observation to revoke consent in case of discomfort and/or disease progression.

In the case of IATs, it is reasonable to infer that they pose additional challenges to patient autonomy and to the informed consent procedure compared to drugs, other non-digital interventions, and mobile-based health-related applications. This is because several IATs (e.g. Doro MemoryPlus 319i phone, GPS SmartSole®) are available over-the-counter as direct-to-consumer products. Therefore, consent from end-users does not occur under medical supervision but is reduced to the acceptance of the device’s terms of service. Notoriously, the terms of service of online applications are rarely read by end-users, making it questionable whether accepting those terms would qualify as informed consent [33]. This problem, which also applies to cognitively healthy users, is exacerbated in the context of older patients with diminished capacity who become users of IATs. Furthermore, several IATs feature artificial intelligence (AI) components such as machine learning algorithms and/or semi-autonomous functionalities. Because such algorithms allow computer systems to learn how to complete tasks in absence of explicit reprogramming, AI-powered IATs are not static but highly dynamic entities. This implies that consenting to the use of an AI-powered IAT now might not necessarily ensure adequate consent in the future since the functionalities and capabilities of the IAT might have evolved. In addition, the incorporation of artificially intelligent components into assistive systems increases the magnitude of ethical significance because it blurs the lines of personal autonomy. In fact, intelligent components blur the separation line between a human user’s decision making and autonomous processing, hence make the attribution of a certain output uncertain. Furthermore, intelligent sensing exacerbates the well-known problem of “digital nudging”. Finally, the technological complexity of several IATs makes it harder for patients and/or their proxy decision makers (e.g. caregivers) to adequately understand what they are consenting to.

While intimately connected with issues of personal autonomy and consent, the problem of introducing an element of human control into closed-loop systems is not entirely reducible to these ethical categories. The moral desirability of introducing veto controls into automated systems is largely independent on whether the end-user has previously consented to their incorporation. For example, veto controls for autonomous systems (including for assistive and rehabilitation robotics) have been proposed as a security-by-default measure that goes beyond individual consent of users [34]. It should be considered, however, that enforcing veto controls in autonomous systems might be difficult, especially in the context of older adults with advanced dementia, as they might have lost their ability to control the system.

The risk of deception is another ethical theme that has been discussed in the literature in the context of pharmacological therapeutics [35]. IATs make the problem of deception more complex by the fact that patients can be deceived not only through receiving false information but also through interacting with conversational agents and/or assistive robots without being able to discern that those agents are not human. This risk, which we may call ‘implicit deception’, is particularly pronounced with humanoid and zoomorphic robots. Even when zoomorphic robots are not explicitly presented to the patients as real pets, their pet-like shape could induce patients to perceive and treat them as biological animals, hence be inherently deceptive. While this problem has already been encountered when using stuffed-toys in dementia care [36], it is exacerbated by intelligent systems’ ability to simulate aspects of the conversational abilities and proxemics of biological agents. Humanoid robots, in particular, add a further layer to this problem as people tend to have unsettling feelings when androids (humanoid robots) and audio/visual simulations closely resemble humans in many respects but are not quite convincingly realistic, a phenomenon known as *uncanny valley* [37].

Our findings also indicate that the moral acceptability of deception is partly dependent on the ethical principle of beneficence. It has been noted that deception, though prima facie wrong, can be justified under certain circumstances with an appeal to promoting the physical and psychological wellbeing of the patient [35]. According to a few interviewees, these circumstances include the use of humanoid or animal-shaped socially assistive robots to reduce agitation and anxiety among older adults with dementia and monitoring their behaviour using integrated sensors and cameras to increase safety. Similar to our findings, a study involving interviews with people with dementia revealed that lies are generally considered to be acceptable by dementia patients if told in their best interest [38]. Similar approaches, however, would require introducing a clear best-interest standard (BIS) [39, 40] for patients with advanced dementia.

Data management, encompassing the processes of data acquisition, storage, processing and sharing is a primary concern when using digital technology [41, 42]. Notoriously, IATs are subject to the typical challenges for personal privacy and medical confidentiality of the broader digital health domain [42]. In particular, many IATs acquire and process data that are not considered strictly medical according to the relevant regulations, but can nonetheless be used to make inference related to health status and behaviour [43]. These data types include swiping behaviour on mobile devices and associated apps, camera-recorded data from home surveillance systems and self-tracked data from wearables that are not classified as medical devices. Our findings illustrate that several grey zones exist in the management of user data for assistive and clinical purposes. These include the security of user-generated datasets and the third-party (re)use of data, especially in light of the risk that insurance companies who access the data may use them against the patients, e.g. by terminating the coverage or increasing the cost of insurance premiums. Overall, the interviewees favoured the promotion of explicit and affirmative consent through opt-in mechanisms when collecting data from patients and, subsequently, the preservation of the patients’ ability to make free and competent decisions about technology use.

With regard to justice considerations, our interviewees favoured inclusive approaches to ensure the fair access to and widespread availability of clinically effective IATs. However, slight divergences emerged in relation to how this can actually be achieved. Several interviewees supported the framework of open development, involving socio-technical approaches such as open-source software and hardware as well as open access to datasets and source codes. Nonetheless, it was observed that the justice-promoting value of open development might, under certain circumstances, conflict with data security or generate liability problems, such the “problem of many hands”, i.e. a gap in a responsibility distribution in a collective setting [44]. Policy solutions were often suggested as complementary measures to ensure fairness in the IAT domain. That is, including IATs into the standard insurance package of seniors with dementia to favour partial or total reimbursement. Our results support the often-advocated proposal to prioritize the reimbursement of those IATs that have demonstrated safety and efficacy and ceteris paribus smaller costs [10, 45]. It is important to emphasize that our study was conducted in countries with different health systems and associated payment models. Italy has a regionally based National Health Service, which provides healthcare services to citizens and residents on a universal basis. Germany has a universal multi-payer health care system paid for by a combination of statutory health insurance (Gesetzliche Krankenversicherung) and private health insurance. In Switzerland, in contrast, there are no free state-provided health services, but private health insurance is compulsory for all residents.

Finally, the general agreement among interviewees regarding the importance of using IATs to complement but not to replace human-delivered care can be interpretable in two ways. First, it might be grounded in a partial mistrust of the technical capabilities of presently available IATs and in a general skepticism that they could, in the short to mid-term future, reach a comparable degree of efficacy, adaptiveness and flexibility as human caregivers. This skeptical attitude was particularly recognizable among those interviewees who were actively operating in the care delivery domain as well as in researchers who experienced translational gaps at the cross-section of technology development and clinical implementation. Second, several interviewees held the normative ethical stance that human-delivered care cannot be replaced by IATs, and that it should not. This ethical stance was often associated with the moral obligations of medical deontology –especially with the principles of beneficence and non-maleficence- and with the importance of preserving a doctor-patient relationship. In relation to this latter point, interviewees argued that this relationship forms one of the foundations of contemporary medical practice and has particular ethical salience. That is, the establishment of a good rapport with the patient is foremost important, where this good rapport should include not only the execution of care tasks, but also adequate communication, mutual trust and empathy. This latter finding seems consistent with previous ethical assessments on assistive robotics [46–50].

## Limitations

Although our study provides important findings that stand to benefit the field of ethics, technology, and aging, it is not without limitations. As evident, this is a study carried out with a small number of experts from three different countries with varying health care systems. In light of the purposive sampling strategy used in the study, our results are neither representative of the expert groups nor the countries from where they were recruited. It cannot be excluded that the study participants discussed ethical issues that they felt the interviewer was interested in capturing. Thus, other ethical issues critical for this field may have remained undiscussed. At the same time, the researcher solicited opinions on cost-related issues and hence, our results related to distributive justice could be in part an artifact of the question posed. Lastly, this study did not interview end-users or their families, thus, our findings are based only on one stakeholder group and not comprehensive of all possible stakeholders engaged in this topic and/or affected by IATs.

## Conclusion

The results of our multi-site qualitative study highlight a multifaceted spectrum of ethical concerns. These findings underline the existence of diverse and substantive ethical challenges associated, among others, with obtaining adequate consent from patients with dementia, respecting their previous wishes as well as current ones inferred from their behaviors, avoiding deception, ensuring fair technology access and ensuring meaningful human contact. The availability of certain IATs on the consumer market might reduce informed consent procedures to the mere acceptance of the product's terms of service. Novel solutions are needed to ensure that end-users understand the technology they are using and its expected benefits and risks (including data collection and sharing). The role of the end-user in controlling his or her own data and, thus, taking a direct role in securing his or her privacy, may be a key to a more user-centered and ethically-aligned deployment of IATs. At the societal level, IATs come with costs that not every end-user can afford. This results in issues of unfair access requiring urgent solution to guarantee that clinically useful IATs are available to those in need. Such policy solution will contribute towards harnessing IATs’ potential in addressing the care needs of the rising aging population. Finally, concerns related to IATs’ possible use as a replacement for human care and whether IAT use constitute deception necessitate further research in understanding personal, ideological, social and cultural concerns, and possible mechanisms of addressing them.

## Supplementary information

**Additional file 1.** Consolidated criteria for reporting qualitative studies (COREQ): 32-item checklist

**Additional file 2.** Original Interview Guide (in German)

**Additional file 3.** Interview Guide (translated in English)

## Data Availability

To preserve the privacy and confidentiality of the research participants, audio records are not made available open access. Non-identifiable supporting materials are deposited as part of the data support services.

## Abbreviations

AI: Artificial intelligence
IAT: Intelligent Assistive Technologies
