# Supplementary material for: Ethical concerns with the use of intelligent assistive technology: findings from a qualitative study with professional stakeholders
Source: BMC Med Ethics. 2019 Dec 19;20:98. doi: 10.1186/s12910-019-0437-z (PMC6924051; doi:10.1186/s12910-019-0437-z)
Supplement: Supplementary file 1 — Additional file 1. Consolidated criteria for reporting qualitative studies (COREQ): 32-item checklist [file 12910_2019_437_MOESM1_ESM.docx]

**Consolidated criteria for reporting qualitative studies (COREQ): 32-item checklist**

**ADD TITLE:**

**Domain 1: Research team and reflexivity**

| 1. | Interviewer/facilitator | In the manuscript (methods section), we have stated that the last author, MI, carried out the interviews. At the time of data collection, MI was a PhD candidate at the University of Basel. As MI is a speaker of German, English and Italian (languages spoken by the interviewees) and has expertise on the ethics of IAT, he was a suitable interviewer for this study. Experience and training of the interviewer is mentioned in the methods section. |
| --- | --- | --- |
| 2. | Credentials | TW and MI have a PhD degree and are trained in qualitative research and, more generally, empirical methodology. RWK has a MD degree, is a chief medical officer in a hospital Department of Geriatric Medicine, and the university chair of geriatrics at the University of Basel. ML is completing a degree in medicine. |
| 3. | Occupation | TW and MI work as researchers (TW is a senior researcher, MI a postdoctoral researcher), ML is a research student, RWK is a geriatrician. ML is a student completing a degree in medicine. Full affiliations are reported in the cover page. |
| 4. | Gender | TW and ML are female, MI and RWK are male. |
| 5. | Experience and training | MI and TW are trained in qualitative methodology. TW is a gerontologist by training and has extensive experience in methodology and elder abuse. RWK has extensive experience in clinical research involving older adults. ML received training in data analysis from MI and TW. |
|  |  |  |
| 6. | Relationship established | There was no professional relationship between the researchers and the participants prior to data collection. Participants knew about our study through a formal invitation to participate in the study. |
| 7. | Participant knowledge of the interviewer | Participants were first informed about the study from an official invitation letter send by MI and RWK. They received information document detailing the study purpose at the time of interview and via email and telephone when interview time and dates were confirmed. |
| 8. | Interviewer characteristics | The interviewer (MI) was completing a PhD in biomedical ethics at the time of data collection. MI is a male. We did not expect any bias. |
|  |  |  |
| **Domain 2: Study design** | | |
| 9. | Methodological orientation and Theory | As reported in the methods section, the methodological orientation of this study was descriptive thematic analysis. |
|  |  |  |
| 10. | Sampling | As reported in the methods section, the study sampling was purposive. |
| 11. | Method of approach | As reported in the methods section, the participants were approached by MI and/or RWK via email. Two participants were informed via telephone communication. |
| 12. | Sample size | As reported in the methods section (Table 1), 20 stakeholders participated in the study. Data breakdown by country, gender and profession is presented in Table 1. |
| 13. | Non-participation | As reported in the methods section (participant recruitment), one participant withdrew from the study after initial enrollment due to health issues. |
|  |  |  |
| 14. | Setting of data collection | Most of the interviews took place at the participants’ workplace and only a few via videoconference upon request of the interviewees. |
| 15. | Presence of non-participants | No |
| 16. | Description of sample | 55% of participants were males, 45% females. Half of them were recruited in Switzerland, 30% in Italy and 20% in Germany. |
|  |  |  |
| 17. | Interview guide | The interview guide was prepared by the authors and experts (acknowledged) were asked to provide their feedback. Since the first few interviews revealed that the questions were appropriate, no changes were made. |
| 18. | Repeat interviews | No |
| 19. | Audio/visual recording | Interviews were tape recorded using a recording device provided by the University of Basel. |
| 20. | Field notes | Field notes were not necessary since all information were recorded in the audio tapes. |
| 21. | Duration | As reported in the methods section, the average duration of the interviews is 33 minutes (range 21 – 55 minutes). |
| 22. | Data saturation | As reported in the methods section, after the interviews and data analysis it was clear that data saturation was reached. |
| 23. | Transcripts returned | No |
|  |  |  |
| **Doman 3: Data analysis** | | |
| 24. | Number of data coders | 3 (MI, TW, ML) |
| 25. | Description of the coding tree | Themes are provided in the paper but not the overall coding tree since the project was larger than what is presented in this paper. |
| 26. | Derivation of themes | As reported in the methods section, themes were inductively derived from the data. |
| 27. | Software | As reported in the methods section, the F4transkript v2 and the MAXQDA Standard software for Windows were used. |
| 28. | Participant checking | As reported in the methods section, all participants were given the opportunity to check the transcripts. Only two participants wished to do so. In both cases, the transcript was approved without modification. |
|  |  |  |
| 29. | Quotations presented | Yes, participants’ quotes are presented and numbered in the results section. |
| 30. | Data and findings consistent | All authors confirm that data and findings are consistent. |
| 31. | Clarity of major themes | In the opinion of all authors, the major themes are clear. Our judgement was corroborated by independent expert assessment (see acknowledgment section). |
|  |  |  |
| 32. | Clarity of minor themes | In the opinion of all authors, the minor themes are clear. Our judgement was corroborated by independent expert assessment (see acknowledgment section). |
