# Supplementary material for: Ethical concerns with the use of intelligent assistive technology: findings from a qualitative study with professional stakeholders
Source: BMC Med Ethics. 2019 Dec 19;20:98. doi: 10.1186/s12910-019-0437-z (PMC6924051; doi:10.1186/s12910-019-0437-z)
Supplement: Supplementary file 2 — Additional file 2. Original Interview Guide (in German) [file 12910_2019_437_MOESM2_ESM.doc]

**Interview Guide**

**Project Title:** Digitalizing Elderly Care in Switzerland: Opportunites and Challenges

***The following open-ended questions will be posed to the study participants and based on their responses further probing questions will be asked.**

**Block 1: Einführung**

1. Wie geht es Ihnen heute?

(Falls Interview findet im Spital statt) Wie sind Sie heute zur Klinik gekommen?

(Falls Interview findet zu Hause statt) Wie läuft Ihr Tag so bisher?

1. Können Sie uns einen typischen Tag von Ihnen beschreiben? Welche Unterhaltung nutzen Sie? (Z.B. Spiele, Fernsehen, usw.)

**Block 2: Betreuungserlebnisse und -bedürfnisse allgemein**

1. Welche Aktivitäten führen Sie im Alltag selber aus?
2. Für welche Sachen brauchen Sie Hilfe? Erleben Sie Einschränkungen in Ihrem Alltag?

*Erstmal auf Antwort warten. Falls die Interview-Person ein Prompt braucht:* *Mobilitätseinschränkungen? Gedächtnis? Physische Unterstützung?*

1. Bei welchen Handlungen brauchen Sie in Ihrem Alltag Unterstützung?

Zum Beispiel: Hilfe beim Essen? Auf Toiletten gehen? Mit den Betreuerinnen zu kommunizieren? Medikamenteneinnahme? Termine einhalten?

*Antwortoptionen: 1) Ja, regelmässig. 2) Ja, aber manschmal. 3) Nein, nie.*

1. Und wer unterstützt Sie (z.B. Spitex, Angehörige) im Alltag?
2. Wie zufrieden sind Sie mit Ihrer Betreuung?

*Promts:*

1 = Sehr zufrieden,  
2 = Zufrieden,  
3= Mittelmäßig,  
4= eher weniger
5= Gar nicht zufrieden

1. Was finden Sie gut? Was finden Sie schlecht? Was sollte man verbessern?
2. Was würde Ihr Alltagsleben erleichtern/verbessern? (Bezug nehmen auf die Antworten von Frage 3)
3. Wie sicher fühlen Sie sich, wenn Sie zu Hause sind?

*Promts:*

1 = Sehr sicher,  
2 = Sicher,  
3= Mittelmäßig,  
4= weniger sicher,  
5= Gar nicht sicher

 Was würde Ihr Sicherheitsgefühl erhöhen?

**Block 3: Technische Hilfsmittel zu Hause & digitale Kenntnisse**

- 1. Sind technische Hilfsmittel bei Ihnen im Einsatz? Wir meinen damit Dinge wie ein Smartphone, ein Tablet oder eine Notfall-Uhr. Wie ist Ihre Erfahrung damit?
  2. (Nur für Smartphone-Users): Finden Sie es schwierig, Ihr Smartphone zu benutzen?

*Promts:*

1 = Sehr schwierig,  
2 = Schwierig,  
3= Mittelmäßig,  
4= weniger schwierig,  
5= Gar nicht schwierig

Wenn ja, was finden Sie besonders schwierig? Die Tasten zu finden? Zu viele Funktionen?

1. Wissen Sie was Apps sind? Verwenden Sie einige? (Nur für Smartphone-Users)
2. Smartphone-User: Welche Apps benutzen Sie am häufigsten? Was ist Ihre Lieblings-App? Verwenden Sie jemals Ihr Smartphone für Gesundheitszwecke?

1. Wenn Sie einverstanden Sie, würden wir Ihnen gerne eine App zeigen.

Technologie 1: 2min Demo + 2min freie Interaktion (Teilnehmerbeobachtung).

1. Wie war Ihre Erfahrung damit? Finden Sie diese App interessant/ einfach zu nutzen? Was würden Sie ändern?
2. Haben Sie je einen Roboter gesehen? Wir zeigen Ihnen gerne einen (demo)

Technologie 2: 2min Demo + 2 min freie Interaktion (Teilnehmerbeobachtung).

1. Wie war Ihre Erfahrung damit? Finden Sie den Teddy interessant/ einfach zu nutzen? Was würden Sie ändern?
2. Würden Sie sich gerne mit dem Teddy sprechen?

*Promts:*

1 = Sehr gerne,  
2 = gerne,  
3= Mittelmäßig,  
4= ungerne,  
5= sehr ungerne

Was würde dafür oder dagegen sprechen?

1. Worüber würden Sie gerne mit dem Teddy sprechen?  Auf welcher Sprache? Was würden Sie gerne von dem Teddy hören?
2. Was denken Sie über diese Technologien, die wir Ihnen gezeigt haben?

**Block 4: Kommunikation**

1. Sie sagten, Sie werden im Alltag von X unterstützt (Frage 6). Wir würden gerne wissen, mit welchen Kommunikationsmitteln Sie mit Ihren Angehörigen / mit Ihren Spitex-Betreuerinnen kommunizieren.

Prompts:

1) Face-to-Face,

2) Telefon/Natel Anruf

3) SMS

4) Whatsapp

5)Skype

6) Andere?

1. Wie effizient finden Sie diese Kommunikation?
   1. = Sehr effizient,  
      2 = effizient,  
      3= Mittelmäßig,  
      4= weniger effizient,  
      5= Gar nicht effizient
2. Würden Sie gerne öfter/besser mit ihnen kommunizieren?

1. Wir zeigen Ihnen gerne ein Produkt, um mit Ihren Angehörigen bzw. Betreuerinnen zu kommunizieren

Technologie 3: 2min Demo + 2 min freie Interaktion (Teilnehmerbeobachtung).

**Block 5: Verbleib zu Hause und kognitive Assistenz**

1. Wie wichtig ist es für Sie, zu Hause zu bleiben anstatt ins Pflegeheim umzuziehen?
   1. = Sehr wichtig,  
      2 = wichtig,  
      3= Mittelmäßig,  
      4= weniger wichtig,  
      5= Gar nicht wichtig
2. Es gibt heute technische Hilfsmittel, welche Ihnen bei der Erinnerung unterstützen können. Vergessen Sie Ihre Medikamente zu nehmen oder den Herd auszuschalten, können Erinnerungen zu Ihnen geschickt werden und Unfälle wie z.B. Stürze verhindert werden. Der Teddy und viele Apps gehören dazu.

Was ist Ihr erstes Gefühl, wenn Sie von solch einer neuen Technologie hören? Neugierde? Angst? Skepsis? Gleichgültigkeit?

1. Wären Sie persönlich bereit, technischen Hilfsmittel wie der Teddy, Sensoren, Mikrophone, Kameras in Ihrer Wohnung zu akzeptieren, um Unfallrisiken zu minimieren?

Prompts:

1 = Sehr bereit,  
2 = bereit,  
3= Mittelmäßig,  
4= weniger bereit,  
5= Gar nichtbereit

Was würde dafür oder dagegen sprechen?

1. Wären Sie bereit, Ihre Wohnung anders zu gestalten, z.B. mit technischen Hilfsmitteln, um länger und unabhängig zu Hause zu bleiben?

1. Ihrer Meinung nach, wer sollte für diese Produkte bezahlen?

1 = Sie
2 = Krankenkasse
3= Spitex
4= Andere

1. Würden Sie gerne einige Produkte, die wir Ihnen gezeigt haben, von Ihrer Krankenkasse bezahlt haben?

1 = Sehr gerne,  
2 = gerne,  
3= Mittelmäßig,  
4= lieber nicht,  
5= überhaupt nicht

1. Würden Sie technische Hilfsmittel als Eingriff in Ihre Privatsphäre einstufen? Würden Sie im Austausch für einen längeren Verbleib im eigenen Zuhause auf einen Teil ihrer Privatsphäre verzichten?
2. Hätten Sie Empfehlungen, um digitale Lösungen für ältere Menschen zu verbessern?
3. Haben Sie sonst noch Fragen?

Vielen Dank für Ihre Zeit.
