# Supplementary material for: Ethical concerns with the use of intelligent assistive technology: findings from a qualitative study with professional stakeholders
Source: BMC Med Ethics. 2019 Dec 19;20:98. doi: 10.1186/s12910-019-0437-z (PMC6924051; doi:10.1186/s12910-019-0437-z)
Supplement: Supplementary file 3 — Additional file 3. Interview Guide (translated in English) [file 12910_2019_437_MOESM3_ESM.doc]

**Interview Guide**

**Project Title:** Digitalizing Elderly Care in Switzerland: Opportunities and Challenges

***The following open-ended questions will be posed to the study participants and based on their responses further probing questions will be asked.**

**Block 1: Introduction**

1. How are you today?

(If interview takes place at the hospital) How did you get to the clinic today?

(If interview takes place at home) How's your day going so far?

1. Can you describe a typical day of yours? What kind of entertainment do you use? (e.g. games, television, etc.)

**Block 2: Care experiences and general needs**

1. Which activities do you carry out yourself in everyday life?
2. What things do you need help for? Do you experience limitations in your everyday life?

Wait for an answer first. If the interviewee needs a prompt:

Mobility restrictions? Memory? Physical support?

1. Which activities do you need support for in your everyday life?

*For instance: Help with eating? Going to the toilets? To communicate with the caretakers? Taking medication? Keeping appointments?*

*Answer options: 1) Yes, regularly. 2) Yes, but sometimes. 3) No, never.*

1. And who supports you (e.g. Spitex, relatives) in everyday life?
2. How satisfied are you with your support?

Promts:

1 = Very satisfied,

2 = Satisfied,

3= Mediocre,

4= rather unsatisfied

5= Not satisfied at all

1. What do you like? What do you think is bad? What should be improved?
2. What would facilitate/improve your everyday life? (Refer to the answers to question 3)
3. How safe do you feel when you're home?

Promts:

1 = Very safe,

2 = Safe,

3= Mediocre,

4= relatively unsafe,

5= Not safe at all

What would increase your sense of safety?

**Block 3: Technical aids at home & digital knowledge**

1. Are you using technical aids? We mean things like a smartphone, a tablet or an emergency clock. What is your experience with them?
2. (For smartphone users only): Do you find it difficult to use your smartphone?

Promts:

1 = Very difficult,

2 = Difficult,

3= Medium,

4= not so difficult,

5= Not difficult at all

If so, what do you find particularly difficult? Find the keys? Too many functions?

1. You know what apps are? Do you use some? (For smartphone users only)
2. Smartphone users: Which apps do you use the most? What is your favorite app? Do you ever use your smartphone for health purposes?
3. If you agree, we would like to show you an app.

Technology 1: 2min Demo + 2 min free Interaction (participant observation)

1. What was your experience with that? Do you find this app interesting / easy to use? What would you change?
2. Have you ever seen a robot? We would be happy to show you one (demo)

Technology 2: 2min Demo + 2 min free interaction (participant observation).

1. What was your experience with that? Do you find the Teddy interesting or easy to use? What would you change?

19. Would you like to talk to the teddy bear?

Promts:

1 = Very gladly,

2 = gladly,

3= medium,

4= rather not,

5= very reluctant

What would be the pros and cons?

1. What would you like to talk to the teddy bear about? In which language? What would you like to hear from the Teddy?
2. What do you think about the technologies we have shown you?

**Block 4: Communication**

1. you said that you are supported by X in everyday life (question 6). We would like to know which means of communication you use to communicate with your relatives and Spitex caregivers.

Prompts:

1) Face-to-face,

2) Telephone/Natel call

3) SMS

4) Whatsapp

5) Skype

6) Others?

1. How efficient do you find this communication?

1 = Very efficient,

2 = efficient,

3= Medium,

4= not so efficient,

5= Not efficient at all

1. Would you like to communicate more often/better?
2. We are happy to show you a product to communicate with your relatives or caregivers

Technology 3: 2min Demo + 2 min free interaction (participant observation).

**Block 5: Staying at home and cognitive assistance**

1. How important is it for you to stay at home instead of moving to a nursing home?

1 = Very important,

2 = important,

3= Medium,

4= not so important,

5= Not important at all

1. Today there are technical aids which can support you in remembering. If you forget to take your medication or switch off the stove, reminders can be sent to you and accidents such as falls can be prevented. The teddy bear and many apps are part of it.

What is your first feeling when you hear about such a new technology? Curiosity? Fear? Skepticism? Indifference?

1. Would you personally be willing to accept technical aids such as the teddy bear, sensors, microphones, cameras in your home to minimize the risk of accidents?

Prompts:

1 = Very ready,

2 = ready,

3= Medium,

4= not so ready,

5= Not ready at all

What would speak for or against?

1. Would you be willing to modify your home, e.g. with technical aids, in order to stay at home longer and independently?
2. In your opinion, who should pay for these products?

1 = you

2 = Health insurance

3= Spitex

4= Other

1. Would you like to have some of the products we showed you paid for by your health insurance company?

1 = Very gladly,

2 = gladly,

3= Medium,

4= not really,

5= not at all

1. Would you classify technical aids as an invasion of your privacy? In exchange for a longer stay in your own home, would you forgo some of your privacy?
2. Do you have any recommendations for improving digital solutions for older people?
3. Any other questions?

Thank you very much for your time.
